# Technique of Brain Surgery

**Published:** 1912-08

**Authors:** Robert Earl

**Affiliations:** St. Paul


### Technique of Brain Surgery

By ROBERT EARL, M.D.
St. Paul

From *The St. Paul Medical Journal*, November, 1911

Nothing new is offered in this paper but the author calls attention to the numerous little details to be observed in intra-cranial surgery.

He describes the Cushing extension attachment for operating tables to facilitate operation on the cerebellum. The large osteoplastic flap is best made with the burrs and forceps devised by Dr. Hudson, of Atlanta.

The author calls particular attention to the dangers of rapid operating and rough handling of brain tissue, and insists on careful hemostasis and gentle handling.

Sub-temporal decompression, as proposed by Cushing, is advised for fractures of the base with symptoms of increasing intra-cranial pressure.

